# Profiling neurotransmitters in a crustacean neural circuit for locomotion

**DOI:** 10.1371/journal.pone.0197781

**Published:** 2018-05-22

**Authors:** Anna C. Schneider, Henriette A. Seichter, Susanne Neupert, A. Maren Hochhaus, Carmen R. Smarandache-Wellmann

**Affiliations:** 1 Zoological Institute, Animal Physiology, Emmy Noether Group, University of Cologne, Cologne, Germany; 2 Zoological Institute, Animal Physiology, University of Cologne, Cologne, Germany; University of Missouri Columbia, UNITED STATES

## Abstract

Locomotor systems are widely used to study rhythmically active neural networks. These networks have to be coordinated in order to produce meaningful behavior. The crayfish swimmeret system is well suited to investigate such coordination of distributed neural oscillators because the neurons and their connectivity for generating and especially for coordinating the motor output are identified. The system maintains a fixed phase lag between the segmental oscillators, independent of cycle period. To further the understanding of the system’s plasticity for keeping the phase lag fixed, we profiled the neurotransmitters used by the Coordinating Neurons, which are necessary and sufficient for coordination of the segmental oscillators. We used a combination of electrophysiological, immunohistochemical, and mass spectrometric methods. This arrangement of methods ensured that we could screen for several specific neurotransmitters, since a single method is often not suitable for all neurotransmitters of interest. In a first step, to preselect neurotransmitter candidates, we investigated the effect of substances known to be present in some swimmeret system neurons on the motor output and coordination. Subsequently, we demonstrated electrophysiologically that the identified synapse between the Coordinating Neurons and their target is mainly chemical, but neither glutamate antagonist nor γ-aminobutyric acid antagonist application affected this synapse. With immunohistochemical experiments, we provide strong evidence that the Coordinating Neurons are not serotonergic. Single-cell MALDI-TOF mass spectrometry with subsequent principal component analysis identified acetylcholine as the putative neurotransmitter for both types of Coordinating Neurons.

## Introduction

Cyclic movements in animals are mainly driven by central pattern generators (CPGs). Both vertebrate and invertebrate models exist in which the generation, coordination, and modulation of the CPGs’ output has been investigated [1]. Although detailed connectomes exist for many of those systems, one needs to know which neurotransmitters and neuromodulators are released by the neurons participating in the generation and coordination of patterned activity in order to understand its plasticity and in-depth function [2].

Limitations of model systems and methods prevent the use of only one method to screen for all possible neurotransmitters of interest. In this study, we tackled the problem by using a variety of methods to identify neurotransmitters in two neuron types that are crucial to the coordination of the crayfish swimmeret system.

**Fig 1:**
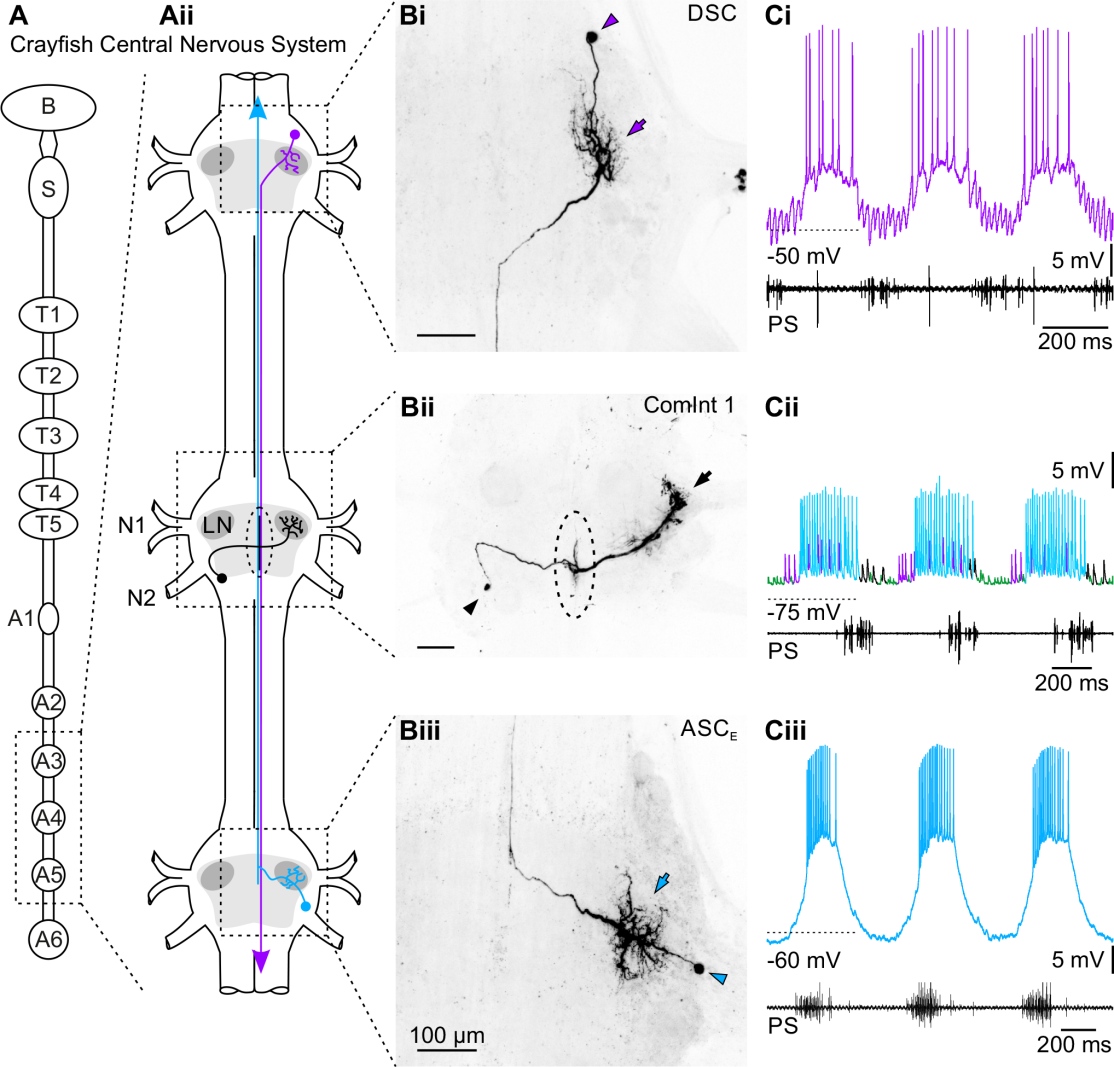
Morphology and activity of the neurons involved in the swimmeret system’s coordination. **A:** Schematic illustration of the crayfish central nervous system (CNS). **Ai:** CNS from the brain (B) to the last abdominal ganglion (A6). Neurons in abdominal ganglia 2 – 5 (A2 – A5) control the swimmeret movement. Bilaterally symmetric copies of both ASC_E_ and ComInt 1 are present in each abdominal ganglion A2 – A5, while DSC can be found bilaterally in A2 – A4. **Aii:** Example of locations and axonal projections of neurons in the coordinating circuit (cyan: ASC_E_, violet: DSC, black: ComInt 1) in a chain of three abdominal ganglia. Arrows on ASC_E_ and DSC axons indicate direction of spike propagation. For simplicity, only ipsilateral copies are depicted. Light grey areas outline a ganglion’s core region containing all neuropils, dark grey areas show the Lateral Neuropil (LN). The dashed circle indicates the area of synaptic contact between presynaptic Coordinating Neurons and postsynaptic ComInt 1. The segmental ganglia are connected via paired connectives; their midline is represented here with a black line. The axons of the Coordinating Neurons project in close proximity to the midline. **B:** Morphology of the three neuron types in the coordinating circuit is shown as whole-mount preparations in dorsal view (scale bar 100μm, applies to all panels). Arrowheads indicate soma; arrows indicate dendritic branching in the Lateral Neuropil. **Bi:** DSC’s soma is located anterior to the first segmental nerve (N1). Dendrites branch in the LN, the axon projects along the midline to posterior ganglia. **Bii:** ComInt 1’s soma is located posterior to N1 on the contralateral side. At the midline, the primary neurite branches anterior and posterior, parallel to the ASC_E_ and DSC axons. Ipsilaterally, the primary neurite branches in the LN. The dashed circle indicates the area of synaptic contact between Coordinating Neurons and ComInt 1. **Biii:** ASC_E_’s soma is located posterior to N1. Dendrites branch in the LN. The axon projects along the midline to anterior ganglia. **C:** Intracellular recordings from different experiments demonstrate the neurons’ activity with respect to the power-stroke (PS) motor activity of their home ganglion. **Ci:** DSC is spiking out of phase with PS activity in its home ganglion. **Cii:** Spikes of the three ipsilateral Coordinating Neurons elicit EPSPs of three distinct sizes in ComInt 1 (see also [14]). In this example, ComInt 1 is recorded in A4. Large EPSPs (cyan) are elicited by the posterior ASC_E_. Medium-sized (violet) come from DSC in the neighboring anterior ganglion. Small EPSPs (green) are in this example elicited by DSC in the most anterior ganglion. **Ciii:** ASC_E_ is spiking in phase with PS activity in its home ganglion. A: Abdominal ganglion; ASC_E_: Ascending Coordinating Neuron; B: Brain; ComInt 1: Commissural Interneuron 1; DSC: Descending Coordinating Neuron; LN: Lateral Neuropil; N1: First segmental nerve; N2: Second segmental nerve; PS: Power-stroke; S: Subesophageal ganglion; T: Thoracic ganglion.

In the crayfish *Pacifastacus leniusculus*, the four pairs of swimmerets are attached to abdominal segments 2 (A2) to A5 and move in an anterior-directed metachronal wave with approximately 25% phase lag between segments [3, 4]. The fictive motor pattern is generated and coordinated without sensory input or descending drive and resembles exactly that observed *in vivo*, which makes it a valuable system to study coordination of CPGs by central connections [5]. The ipsilateral coordinating circuit comprises three neurons per hemiganglion (Fig 1), which are necessary and sufficient to maintain the phase lag: The Ascending (ASC_E_, Fig 1Biii) and Descending Coordinating Neuron (DSC, Fig 1Bi), and the Commissural Interneuron 1 (ComInt 1, Fig 1Bii) [6]. The Coordinating Neurons encode information about their home ganglion’s motor output as bursts of spikes [7–10]. ASC_E_ is active in phase with power-stroke (PS) motor neurons (Fig 1Ciii), and projects along the midline to anterior target ganglia (Fig 1Aii and 1Biii). DSC is active in antiphase to PS (Fig 1Ci) and projects along the midline to posterior target ganglia (Fig 1Aii and 1Bi). Activity of the Coordinating Neurons is able to modulate the phase lag and number of recruited motor units of their target ganglia [7, 11, 12].

ComInt 1’s soma is located contralateral in each hemiganglion, whereas the synapses to the CPG in the Lateral Neuropil (LN) are on the ipsilateral side (Fig 1Aii and 1Bii). Its primary neurite crosses the midline, where it sends off processes in the anterior and posterior direction to pick up coordinating information (area indicated by dashed circle in Fig 1Aii and 1Bii) in the form of fast and brief excitatory postsynaptic potentials (EPSPs) whose sizes are graded by segmental distance (Fig 1Cii) [13–15]. Each ComInt 1 receives information from three ipsilateral Coordinating Neurons. Depending on the segment it is localized in, it receives in A2 and A5 coordinating input from only one type of Coordinating Neuron (ASC_E_ or DSC, respectively), or, in the middle ganglia (A3 and A4), from both types (ASC_E_ and DSC) [14].

To preselect neurotransmitter candidates, we focused on substances known to be present in the crustacean ventral nerve cord. Motor neurons release glutamate or γ-aminobutyric acid (GABA) [16–18]. Serotonergic fiber bundles have been identified in all abdominal ganglia in lobster [19]. Cholinergic agonists both activate and modulate the fictive swimmeret motor pattern [20–23].

For further selection, we first analyzed whether these neurotransmitters affected the motor output. In order to determine whether the substance impacted the coordinating circuit, we paid special attention to changes in the coordination pattern when substances were applied to only a single ganglion. Second, we demonstrated that the synapse between the Coordinating Neurons and ComInt 1 is chemical and, therefore, could be a target for the neurotransmitters. Third, we profiled each of the preselected neurotransmitters with a different approach, because a single method was not reliable for all of those neurotransmitters. For instance, GABA and glutamate agonists and antagonists were shown to be very effective in crustaceans by electrophysiological approaches [16, 24–27]. However, this method has not been effective for identification of 5-HT effects on the system in the past [5]. For studying the occurrence of 5-HT, immunohistochemistry was used previously to identify 5-HT-positive neurons and fiber bundles in lobster [19]. Another neurotransmitter candidate, acetylcholine (ACh), is more difficult to identify in crustaceans. Although ACh application can induce rhythmic activity in crayfish walking leg motor neuron pools, and focal application even elicits differential changes in membrane potential in those motor neurons [28], ACh antagonists have a complex profile in crustaceans [29] and polyclonal antibodies against choline acetyltransferase, the synthetic enzyme, have failed in this system [6]. Therefore, we used mass spectrometry to investigate the presence of ACh in the Coordinating Neurons.

## Materials and methods

### Animals and dissection

All experiments were carried out in isolated abdominal nervous systems of the signal crayfish *Pacifastacus leniusculus*, Dana 1852, of both sexes. Animals were acquired from a local fisherman in the river Wupper, Germany, and kept in appropriate water tanks at 14°C. Dissection of the nervous system was done according to Seichter and colleagues [30]. Briefly, animals were anesthetized in ice, exsanguinated, decapitated, and the nervous system carefully dissected from thoracic ganglion 4 (T4) to the terminal abdominal ganglion (A6). The first segmental nerves (N1) of the abdominal ganglia A2 – A5 were preserved as long as possible for recording, the other nerves were cut short. The ganglia chain was pinned out dorsal side up in a petri dish lined with Sylgard (Dow Corning, Midland, Michigan, USA) and filled with normal saline (concentrations in mM: 195.0 NaCl, 5.4 KCl, 2.6 MgCl_2_, 15.8 CaCl_2_, buffered with 1 M Trizma base and 0.5 M maleic acid at pH 7.4 in aerated saline). All ganglia were dorsally desheathed using fine scissors to facilitate uptake of chemicals and to ensure better penetration of antibodies.

### Electrophysiology

Activity of power-stroke (PS) motor neurons was recorded with stainless steel pin electrodes from the posterior branch of N1 on both sides of ganglia A2 to A5. Nerve and recording electrode were insulated from the bath with petroleum jelly. The reference electrode was placed in the bath close to the recording electrode. If preparations did not show spontaneous activity, it was induced with 2 μM – 4 μM carbachol (Sigma, St. Louis, Missouri, USA). Sharp microelectrodes pulled from borosilicate capillaries (o.d. 1.0 mm, i.d. 0.5 mm) filled with 1% dextran Texas Red (dTR; Invitrogen, Carlsbad, CA, USA) in 1 M KAc + 0.1 M KCl (electrode resistance 30 MΩ – 45 MΩ) were used to record intracellularly from the Commissural Interneuron 1 (ComInt 1) at the ganglion midline, or the Coordinating Neurons in the LN. After impaling a neuron, it was physiologically identified by its spiking activity and effect on the motor output. After the experiment, its identity was confirmed by morphology with iontophoretic dye injection for approximately 15 min using 250 ms +1 nA pulses at 2 Hz.

Extracellular electrodes were connected to a differential amplifier (Model MA 102, Animal Physiology Electronics Lab, University of Cologne, Cologne, Germany). Intracellular signals were amplified with an intracellular amplifier SEC-05X (npi Electronic Instruments, Tamm, Germany). All signals were digitized with a Digidata 1400 (Molecular Devices; Sunnyvale, CA, USA) and recorded with Clampex (Molecular Devices).

During electrophysiological experiments, preparations were constantly perfused with saline. For blocking experiments, we made a large petroleum jelly well around ganglion A4 and perfused it with one of the following solutions: low Ca^2+^ / high Mg^2+^ saline (concentrations in mM: 118.0 NaCl, 5.33 KCl, 52.0 MgCl_2_, 2.4 CaCl_2_, buffered with 10 mM Trizma base and 4.7 mM maleic acid at pH 7.4 in aerated saline), 6 μM – 120 μM 6,7-dinitroquinoxaline-2,3-dione (DNQX, glutamate antagonist, BioTrend, Cologne, Germany), 100 μM – 5 mM picrotoxin (PTX, GABA antagonist; Sigma), or 0.5 mM - 1 mM serotonin (5-HT, Sigma). For wash out, we perfused the treated segment with normal saline for at least one hour. Focal application of drugs was mediated by a PDES 2L pressure ejection system (npi Electronic Instruments) connected to a capillary positioned close to ComInt 1’s recording site at the ganglion midline. 0.8 mM DNQX was applied for 4 s each 5 s, 5 mM PTX was applied for 10 s each 20 s with 0.5 bar. When we had to induce rhythmic activity with carbachol, we mixed the drugs into saline with the same carbachol concentration to exclude effects which could have been caused by a lack of cholinergic agonists.

### Analysis of electrophysiological data

Coordination between segments was analyzed as follows: The beginning of each PS burst in A5 served as reference to calculate phase lags. Phase lag was calculated as the latency from beginning of the reference burst to beginning of another burst, e.g. PS4, divided by cycle period. Means and SD of at least 10 consecutive bursts were used in this analysis. To reveal rhythmic modulation of apparently tonic PS activity, we rectified and smoothed the voltage recordings with Spike2 (CED, Cambridge, UK), using a time constant of 0.1 s for smoothing.

The size and shape indices of the EPSPs in ComInt 1 were measured with Clampfit (Molecular Devices). First, at least 30 EPSPs were triggered on a specific ASC_E_ or DSC recording and averaged. From this, we used the build-in Clampfit tool “Statistics” to calculate the EPSP shape indices.

For the blocking experiments we averaged and compared amplitudes and spike indices of at least 10 EPSPs under control condition, and at the beginning and end of DNQX and PTX application using custom-written MATLAB (MathWorks, Natick, MA, USA) scripts. For simplification, we focus on the large and medium EPSPs in this analysis as they come from different types Coordinating Neurons (ASC_E_ and DSC).

“N” denotes number of experiments/animals; “n” the number of trials. Results from different treatments were compared in MATLAB with repeated-measures ANOVA (RM-ANOVA) with Tukey’s post-test, or paired t-test if only two dependent conditions were tested. Shape indices of EPSPs from ASC_E_ and DSC were compared with unpaired t-test. If the assumption of sphericity was violated for RM-ANOVA, Greenhouse-Geisser (GG) correction was used. Data and results of the statistic tests can be found in the supplementary spreadsheets. Because we could not detect any obvious differences in results obtained from experiments with bath or focal application we pooled those results for statistical analyses.

### Neuron labeling by dye injection and immunohistochemistry

We used whole-mount preparations of abdominal ganglia A2 – A5 to examine if Coordinating Neurons contained 5-HT. Before immunohistochemistry, Coordinating Neurons were iontophoretically filled with 1% dTR (dextran Texas Red) for at least 30 min using 250 ms +1 nA pulses at 2 Hz. For dye transportation back into the soma and along its axon, preparations were kept at 4°C for up to three days.

After that, ganglia were fixed for 2 h at room temperature in Roti Histofix (Carl Roth, Karlsruhe, Germany) or 4% paraformaldehyde (Merck, Darmstadt, Germany) + 0.5% glacial acetic acid in 0.1 M phosphate buffered saline (PBS; concentrations in M: 0.1 Na_2_HPO_4_, 0.01 NaH_2_PO_4_, 0.15 NaCl). After fixation, samples were washed 3x10 min in PBS. For antibody labeling, ganglia were first preincubated in PBS containing 1% Triton-X-100 (PBST) and 5% normal goat serum + 0.1% NaAc (NGS) and then incubated for 12 h – 36 h at 4°C using rabbit anti-5-HT (Sigma) diluted 1:400 in PBST-NGS. Subsequently, ganglia were washed 3x2 h in PBST and incubated overnight at 4°C in goat anti-rabbit Alexa Fluor 488 conjugated (abcam, Cambridge, UK), diluted 1:200 in PBST-NGS. Afterwards, ganglia were washed 4x1 h in PBS, dehydrated in ascending ethanol concentrations, and mounted on microscope slides in methylsalicylate (Carl Roth).

Samples were visualized using a confocal laser scanning microscope (LSM 510 Meta, Zeiss, Oberkochen, Germany) with 10x (Plan-APOCHROMAT 10x/0.45) or 40x (Plan-NEOFLUAR, 40x/1.30 oil) magnification. The microscope was equipped with a primary dichroic beamsplitter HTF 488/543 for excitation. Emission of Alexa Fluor 488 was filtered by a 505–530 nm band pass filter, emission of dTR by a 650 nm long pass filter. Samples were scanned as z-stacks (10μm step size) of which maximum intensity or depth-coded projections were generated with ZEN 2011 black edition (Zeiss). Brightness and contrast were adjusted with Photoshop (CS 5, Adobe System, San Jose, CA, USA). Videos were prepared with ImageJ/FIJI [31, 32].

### Sample preparation for single cell MALDI-TOF mass spectrometry

For cell identification, Coordinating Neurons were labeled intracellularly with 1% dTR for up to two hours and processed immediately if the soma was visible, or after overnight incubation at 4°C to allow additional dye diffusion. Motor neurons were backfilled via N1 with 1% dTR overnight. For single cell dissection, abdominal ganglia were first washed in single cell saline (concentrations in mM: 46 NaCl, 4.0 KCl, 7.5 CaCl_2_ at pH 7.4) adapted from Jullien and Ripplinger [33]. Then the ganglion was fixed with microneedles in a preparation dish and covered with fresh single cell saline containing 33% glycerol to stabilize the fluorescent dye. Under a stereo fluorescence microscope (SteREO Lumar V12, Zeiss) equipped with a Lumar 43 HE Cy3 filter, the soma was isolated and dissected from the ganglion using ultra-fine scissors, and transferred manually with a glass capillary to a stainless steel sample plate for matrix assisted laser desorption/ionization (MALDI)—time of flight (TOF) mass spectrometry. The saline around the soma was completely removed with the same glass capillary. Without any additional rinsing steps, the soma was covered with 10–20 nl saturated alpha-cyano-4-hydroxycinnamic acid (CHCA) as matrix diluted in 50% methanol/water in a ratio of 1:2.

### MALDI-TOF mass spectrometry

Mass spectra were acquired under manual control in positive ion mode on an UltrafleXtreme TOF/TOF mass spectrometer (Bruker Daltonics, Billerica, MA, USA). The instrument settings were optimized for mass ranges of m/z 0 – 300 Da and calibrated using a mixture of synthetic GABA and acetylcholine, respectively. Laser fluency was adjusted to provide optimal signal-to-noise ratio. MS/MS was performed with LIFT technology by an acceleration set at 1 kV. The number of laser shots used to obtain a spectrum varied from 1000 to 5000 depending on ion signal quality. The putative acetylcholine ion signal was verified using MS/MS fragmentation pattern and compared using fragmentation data provided by Scripps METLIN Center of Metabolomics [34]. All data obtained in these experiments were processed with the FlexAnalysis 3.4 software package (Bruker Daltonics).

To compare MALDI-TOF data of single ASC_E_, DSC, and motor neuron preparations, we used ClinPro Tools software 3.0 (Bruker Daltonics) for comparison of signal intensities in a mass range of m/z 100–300. For that, MALDI MS data were uploaded and the following settings for PCA analysis were used: resolution 800, top hat baseline at 10.0% minimal baseline width, enable smoothing at 0.1 width (m/z) with 5 cycles using Savitzky-Golay filter type, recalibration of 1000 ppm maximal peak shift, 30% match to calibrant peaks, and a peak picking on total average spectrum at 4.00 signal to noise threshold.

## Results

Although neuronal connectivity, which is sufficient to drive the swimmeret rhythm, is identified [10, 14, 15, 35, 36], potential neurotransmitters of central neurons in the swimmeret system have not yet been investigated. In this study, we screened for neurotransmitters of the Coordinating Neurons by combining electrophysiological, immunohistochemical, and mass spectrometric approaches.

### Neurotransmitters affecting the motor output

The activity of Coordinating Neurons modulates the motor output of their target ganglia by increasing or decreasing the spiking frequency and number of recruited motor neurons, and / or altering the phase relationship between segments [7, 11, 12]. Coordinating information is integrated by one neuron in each hemisegment: Each spike from the Coordinating Neurons elicits a distinct EPSP in their target ComInt 1, which, in turn, is electrically coupled to one of the segment’s CPG neurons [14, 15]. This information transfer has to be reliable in order to maintain the phase relationship between segments. One step towards a deeper understanding of this connectivity is the identification of the neurotransmitter involved in transferring this information. Because of the vast abundance of neurotransmitters, we needed a preselection in the swimmeret system for our screening purposes. Hence, we first identified neurotransmitters and neuromodulators that changed the swimmeret system’s motor activity, specifically the phase relationship between segments, because they could possibly act by influencing the information transmission between the Coordinating Neurons and ComInt 1.

ACh is present in many sensory neurons, and cholinergic agonists are known to activate and modulate the swimmeret rhythm and have been used for decades to stimulate quiescent preparations [20, 37]. Furthermore, application of cholinergic agonists to only one half of a split-bath preparation changes the phase relationships between the segments at the boundary [23]. Therefore, ACh was one of our neurotransmitter candidates. To find further possible neurotransmitters that influenced the coordination between segments, we used an electrophysiological approach. For this, we focused on neuroactive substances that have been identified in other neurons in the crustacean ventral nerve cord. Glutamatergic and GABAergic ascending interneurons have been described in the terminal ganglion A6 [38–40]. Additionally, glutamate and GABA are synthesized by excitatory and inhibitory swimmeret motor neurons, respectively [16–18]. Furthermore, those motor neurons share some characteristics with the Coordinating Neurons, e.g. soma location, area of dendritic arborization, phase of activity, and innervation by the CPG (cf. Fig 1 and [41]). With respect to morphological similarities, Coordinating Neurons are also present in the locations of the four serotonergic fiber bundles identified in lobster [19], allowing the possibility that they contain 5-HT and are part of those fiber bundles. Hence, glutamate, GABA, and 5-HT were considered neurotransmitter candidates in addition to ACh.

**Fig 2:**
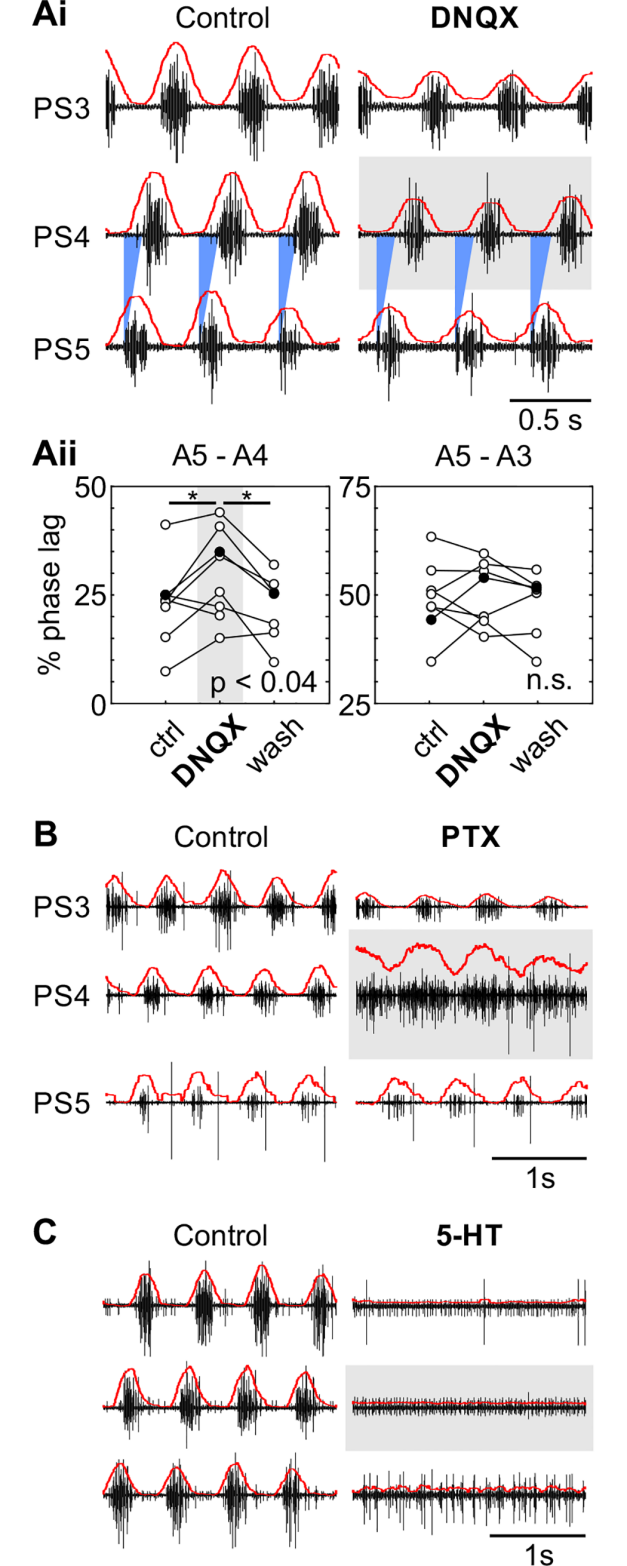
Glutamate antagonists, GABA antagonists, and 5-HT affected intersegmental coordination and rhythmic activity. Raw PS recordings are shown in black, rectified and smoothed recordings are superimposed as red lines. The pharmacological agents were applied to one segment only, shown here with a grey box. **A:** Phase lag of power-stroke (PS) activity in ganglion A4 without and with DNQX application (grey box). **Ai:** PS activity in three ganglia of the same preparation. In control condition (left) all motor bursts were properly coordinated. When the glutamate antagonist DNQX (6μM) was perfused over ganglion A4 (grey box) its PS was delayed with respect to PS5 (right). Blue triangles indicate 25% phase lag with respect to PS5. **Aii:** Mean phase lag of PS activity in ganglion A4 and A3 before, during, and after application of the glutamate antagonist DNQX to A4 (grey background) in eight different preparations. Individual experiments are connected with lines. During DNQX perfusion, the phase lag of A4 increased in 6 of 8 experiments (F(2,12) = 8.095, p < 0.01). After wash-out, the phase lag recovered in 5 of 7 experiments. No rhythmic activity could be recorded in 1 of 8 experiments after washing. The phase lag between the untreated ganglia A5-A3 did not change significantly. Filled circles highlight data from the experiment in Ai. See also S1 Table. **B:** PS activity recorded from three different ganglia of the same preparation without and with PTX application. In control condition (left), the bursting activity in each segment is clearly distinguishable. The coordinated activity starts in the posterior segment and the other follow with approximately 25% phase lag. When the GABA antagonist PTX (400μM) was perfused over ganglion A4 (grey box) its PS became tonic (right). This tonic activity was still modulated on a cycle-by-cycle basis (red line). Coordination between untreated ganglia was unaffected. **C:** PS activity recorded from three different ganglia of the same preparation without and with 5-HT application. In control condition (left), the bursting activity in each segment is clearly distinguishable. The coordinated activity starts in the posterior segment and the others follow with approximately 25% phase lag. When 5-HT (500 μM) was perfused over ganglion A4 (grey box) bursting activity ceased in all ganglia (right). All data and statistics for these experiments are in S1 Table.

First, we tested whether any of those candidates affected the coordination and the motor output of the swimmeret system. For this, we recorded the motor activity of the isolated abdominal ganglia chain and perfused only ganglion A4 with the glutamate antagonist DNQX (6 μM– 60 μM), the GABA antagonist PTX (100 μM), or 5-HT (0.5 μM– 1 μM) (Fig 2). Both antagonists have been shown to be effective in crustaceans, also specifically in crayfish [16, 42–44].

The phase lag between neighboring ganglia was approximately 25% in control condition. In comparison, when DNQX blocked glutamatergic transmission in A4, the phase lag was significantly greater in that ganglion with respect to A5 (Fig 2A; S1 Table). PS activity of the perfused ganglion was delayed: the phase lag increased in 6 of 8 experiments (Fig 2Aii). DNQX applied specifically on A4 did not affect the phase lag between the untreated ganglia A5-A3 (Fig 2Aii). Some experiments seemed to reflect in A3 a change in phase lag seen in A4, which can be explained by the closed loop of coordinating information transfer in the swimmeret system. We hypothesized that DNQX might block synaptic transmission from Coordinating Neurons to ComInt 1, disturbing the coordination between segments.

In contrast to DNQX, perfusion of PTX over A4 (N = 6, Fig 2B) resulted in tonic motor activity with recruitment of larger units in that segment. Similar results were reported by Sherff and Mulloney [25] for application of PTX to the whole chain of abdominal ganglia. Rectifying and smoothing the raw recording (red lines in Fig 2) revealed that the tonic activity was still modulated on a cycle-by-cycle basis. We therefore assume that PTX application disinhibited motor neurons, while the CPG continued to oscillate. Because of the increased tonic activity, we were unable to calculate phase relationships of the PTX-treated ganglion.

When applying 5-HT to A4 (N = 6, Fig 2C), PS activity became either tonic on all recorded motor nerves (4 of 6 experiments), or only in the treated ganglion A4 (2 of 6 experiments). After washing with normal saline, rhythmic activity recovered, regardless of the applied substance. Because of their ability to corrupt the coordinated motor pattern, glutamate, GABA, and 5-HT qualified as neurotransmitter candidates in addition to ACh and were further investigated.

### The synapse from the Coordinating Neurons onto ComInt 1 is mainly chemical

**Fig 3:**
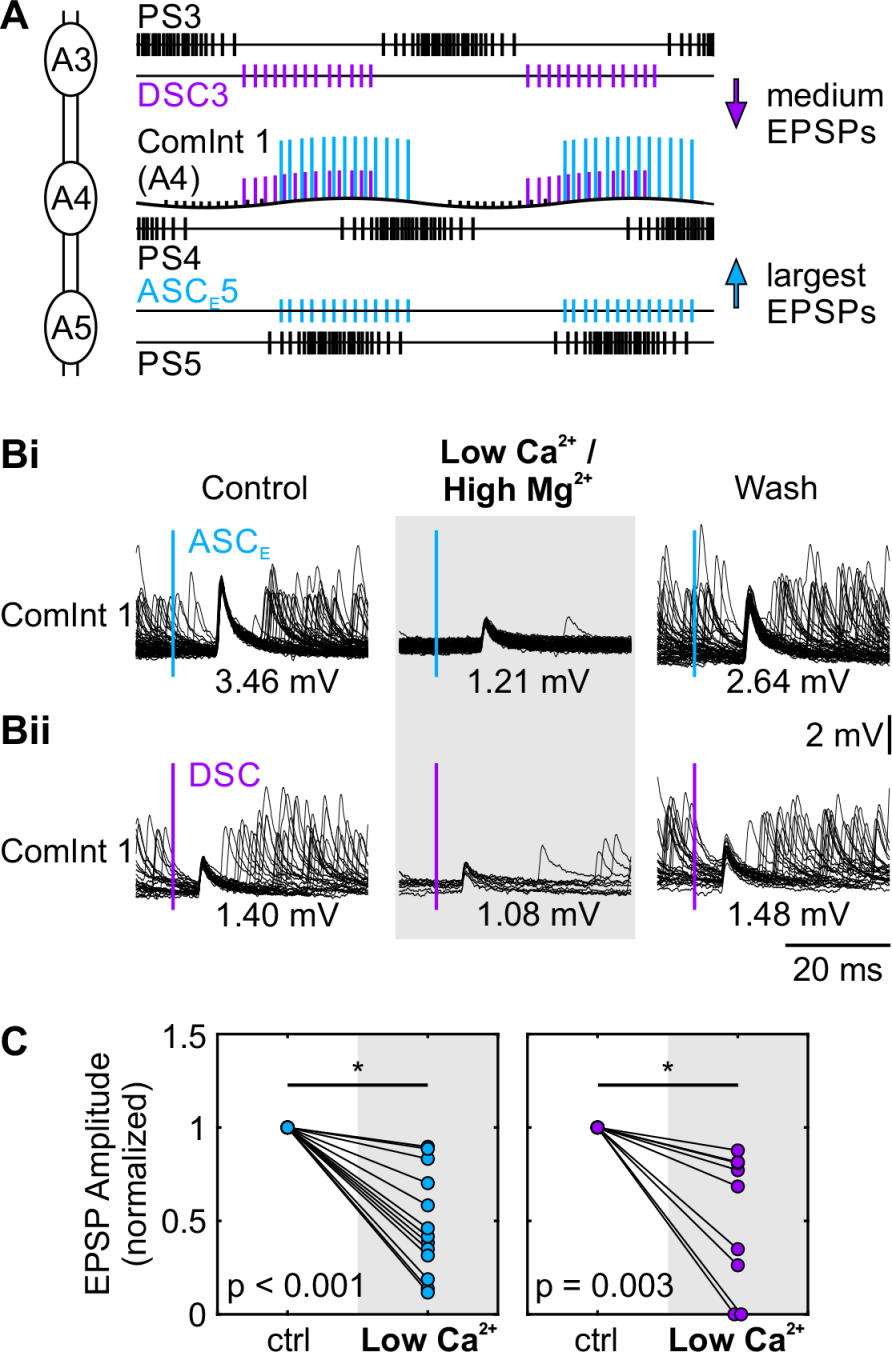
The synapse between Coordinating Neurons and ComInt 1 is mainly chemical. **A:** Schematic of activity in a chain of three connected ganglia and the input ComInt 1 receives in the middle ganglion. PS motor neuron activity is shown in black, ASC_E_ in cyan, and DSC in violet. ComInt 1 is depicted as intracellular recording where color-coded EPSPs elicited by the posterior ASC_E_ (from A5) are the largest, and those elicited by the anterior DSC (from A3) are medium-sized. Black EPSPs come in this example from the most anterior DSC in A2. The rhythmic activity starts with a burst of action potentials of PS motor neurons in the most posterior ganglion (A5), the anterior ones follow with a phase lag of approximately 25%. Because of this phase lag, EPSPs elicited by spikes from the posterior neighboring ASC_E_ (A5, in phase with PS) and anterior DSC (A3, out of phase with PS) arrive almost simultaneously in ComInt 1 in A4 (see also [14]). **B:** Overdraw of one ComInt 1 recording in A3 when triggered on ASC_E_ (**Bi**) or DSC spikes (**Bii**) from neighboring ganglia in normal saline (control, wash) and low Ca^2+^ / high Mg^2+^ saline. The time of the coordinating spike used for triggering the EPSP overdraw is indicated by the colored bar. Values in mV give average EPSP amplitude. **C:** Change in the normalized amplitude of both large (left panel, cyan) and medium sized (right panel, violet) EPSPs recorded in ComInt 1 in normal saline (ctrl) and lowCa^2+^ / high Mg^2+^ saline (N = 13). Amplitudes were significantly reduced in lowCa^2+^ / high Mg^2+^ saline (paired t-test). A: Abdominal ganglion; ASC_E_: Ascending Coordinating Neuron; DSC: Descending Coordinating Neuron; ComInt 1: Commissural Interneuron 1; PS: Power-stroke. All raw data and statistics for these experiments are in S2 Table.

After the preselection of neurotransmitter candidates, we focused our investigation on the synapse between the Coordinating Neurons and ComInt 1. First, we had to examine whether the synapse is chemical or electrical. Each spike of the Coordinating Neurons elicits a distinct and fast EPSP in their target ganglia’s ComInt 1. Largest EPSPs are elicited by the Coordinating Neurons originating in immediate neighboring ganglia, and EPSPs elicited by the posterior ASC_E_ are always larger than those of the anterior DSC (Fig 3A and 3B) [14]. Therefore, the largest EPSPs in A4’s ComInt 1 are elicited by ASC_E_ spikes originating in A5 (Fig 3A), medium EPSPs by DSC spikes in the neighboring A3. Smallest EPSPs are always elicited from the most distal Coordinating Neuron, in this case DSC in A2

Because EPSP amplitude strongly depends on the recording site, which could differ between experiments, we refer to the EPSPs elicited by different Coordinating Neurons as large, medium, and small, rather than giving the absolute amplitude.

We replaced the saline in the bath around one ganglion with low Ca^2+^ / high Mg^2+^ saline to block neurotransmitter release at chemical synapses (N = 13), while the activity of the neighboring segments was preserved. Therefore, ASC_E_ and DSC from the neighboring ganglia were still active and sent spikes to the ganglion bathed in low Ca^2+^ / high Mg^2+^ saline [8]. In all cases, EPSP size in ComInt 1 was significantly reduced (Fig 3B and 3C, S2 Table). In four of these experiments, we subsequently replaced the low Ca^2+^ / high Mg^2+^ saline with normal saline and demonstrated a recovery in EPSP size (Fig 3B). The average reduction of EPSP amplitude by 50% in low Ca^2+^ / high Mg^2+^ saline and subsequent recovery shows that the synapse between Coordinating Neurons and ComInt 1 has a chemical component. Electrical connections between neurons are unaffected by low Ca^2+^ / high Mg^2+^ saline and might account for the residual, diminished EPSPs. The actual proportion of the chemical component could be underestimated in our experiments because the calcium concentration we used in the low Ca^2+^ / high Mg^2+^ saline was shown to be not as effective as other concentrations [45].

As a consequence, the EPSPs in ComInt 1 have to be elicited by neurotransmitter release. ASC_E_ and DSC are two distinct Coordinating Neurons, therefore it is feasible that they might use different neurotransmitters at the synapse to ComInt 1. Interestingly, EPSP shape indices, i.e. rise-time and half-width, did not differ between those elicited by ASC_E_ or by DSC, regardless of ComInt 1 location (i.e. ComInt 1 located in A3 or A4; see Table 1, S3 Table). Therefore, we hypothesized that ASC_E_ and DSC use the same neurotransmitter.

**Table 1:** EPSP shape indices (in milliseconds) from Coordinating Neurons recorded in ComInt 1 in ganglia A3 and A4.

	ComInt 1 in A3		ComInt 1 in A4	
	ASC_E_4 (larg. EPSP)	DSC2 (med. EPSP)	ASC_E_5 (larg. EPSP)	DSC3 (med. EPSP)
*N*	*7*	*5*	*7*	*8*
***Rise-time***				
Mean ± SD	0.8 ± 0.1	0.7 ± 0.1	0.8 ± 0.1	0.8 ± 0.1
*Range*	*0.61–0.9*	*0.59–0.89*	*0.65–1.06*	*0.62–1.1*
***Half-width***				
Mean ± SD	2.9 ± 0.9	3.1 ± 0.9	2.6 ± 1.3	2.4 ± 0.9
*Range*	*1.95–4.72*	*2.32–4.68*	*1.45–5.76*	*1.43–4.58*

There was no difference between shape indices of EPSPs elicited by ASC_E_ or DSC (t-test not significant, p > 0.19). See also S3 Table.

Values are given in milliseconds. *N*: Number of experiments.

### Coordinating Neurons do not release glutamate or GABA at their synapse to ComInt 1

After confirming that the synapse between the Coordinating Neurons and ComInt 1 is chemical, we investigated whether Coordinating Neurons released the same neurotransmitters as the motor neurons (glutamate and GABA). This seemed a valid hypothesis because DNQX application delayed the motor bursts of the affected ganglion and PTX application corrupted the coordinated motor activity. We recorded intracellularly from ComInt 1 and applied a glutamate antagonist or GABA antagonist either to the bath or focally on the neuron. We expected that, if these antagonists would be effective, the EPSP shape and size recorded in ComInt 1 should change.

**Fig 4:**
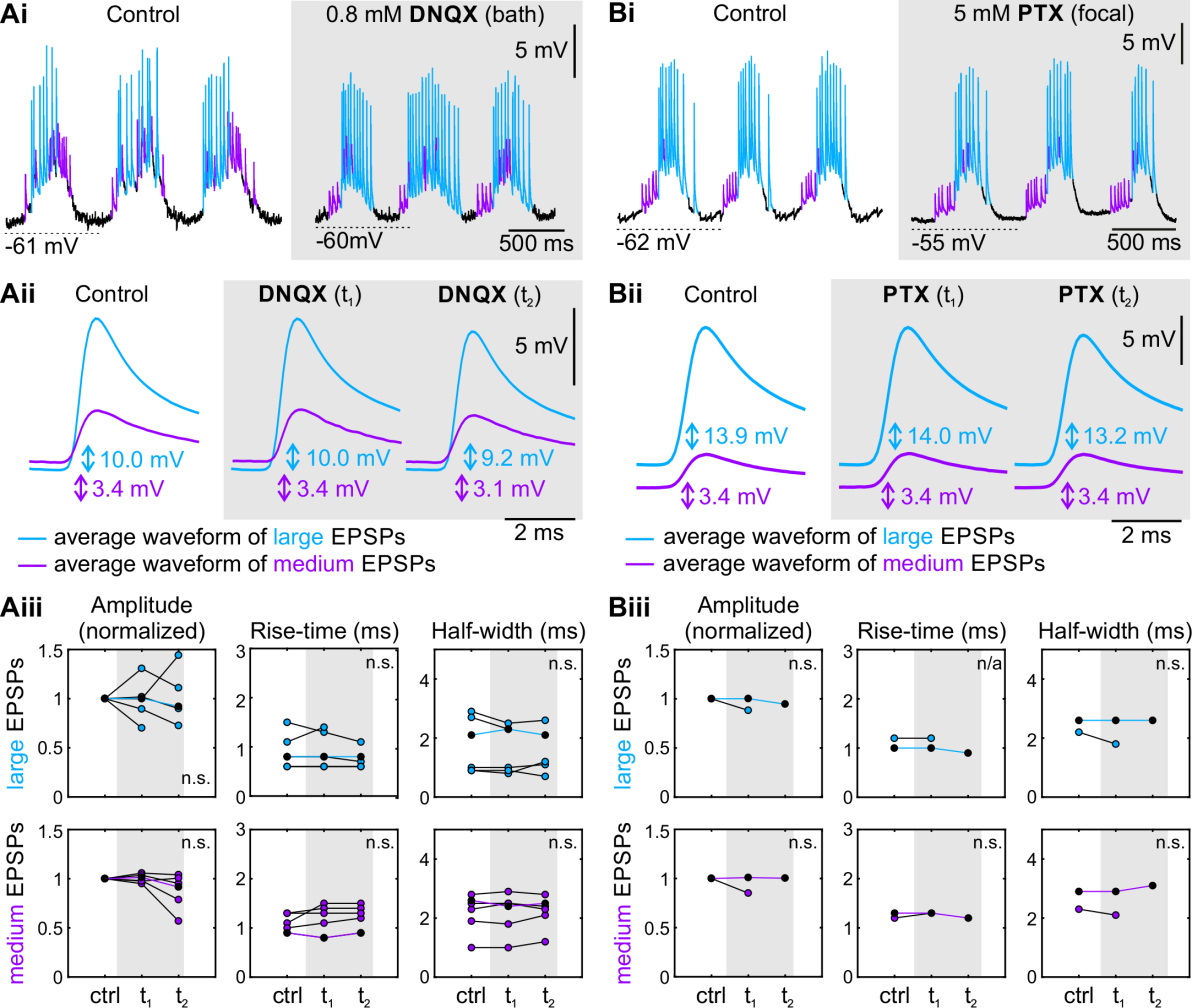
Glutamate antagonists and GABA antagonists did not change EPSP amplitude or shape in ComInt 1. Largest and medium-sized EPSPs are shown in cyan and violet, respectively. See also S4 Table. **Ai:** Intracellular recording of ComInt 1 in normal saline and during bath application of 0.8 mM DNQX (glutamate antagonist). **Aii:** Average waveform of largest and medium-sized EPSPs from one experiment (**Ai**) in normal saline and during DNQX application (grey background) at the beginning (t_1_ = 14 min) and later (t_2_ = 30 min) in the recording. Shape and amplitude of both EPSP types did not differ between control and DNQX application. **Aiii:** Data for individual experiments with DNQX application (grey box). Individual experiments are connected by lines. Data points for the experiment shown in Ai and Aii are highlighted by black fills with colored lines. Neither the absolute amplitude of EPSPs [F(2,8) = 0.017, p = 0.982 for large EPSPs; F(1.12,5.62) = 5.803, p = 0.053 for medium EPSPs], nor the normalized amplitudes were significantly different. No significant difference was found for the rise-time [F(1.04,4.18) = 1.652, p = 0.268 for large EPSPs; F(1.11,5.57) = 1.947, p = 0.219 for medium EPSPs] or half-width [F(2,8) = 0.167, p = 0.849 for large EPSPs; F(2,10) = 0.156, p = 0.857 for medium EPSPs]. **Bi:** Intracellular recording of ComInt 1 in normal saline and during focal application of 5 mM PTX (GABA antagonist). **Bii:** Average waveform of largest and medium-sized EPSPs from one experiment in normal saline and during focal PTX application (grey background) at the beginning (t_1_ = 0 s) and later (t_2_ = 160 s) in the recording. Shape and amplitude of both EPSP types did not differ between control and PTX application. **Biii:** Data for individual experiments with PTX application (grey box). Individual experiments are connected by lines. Data points for the experiment shown in Bi and Bii are highlighted by black fills with colored lines. Paired t-test p ≥ 0.5 for amplitude, rise-time, and half-width of large and medium EPSPs shows no statistical difference in the shape indices. All data and statistics for these experiments are in S4 Table.

Neither bath nor focal application of the glutamatergic antagonist DNQX affected EPSP amplitude, rise-time, or half width in ComInt 1 (Fig 4A; S4 Table). ComInt 1’s membrane potential did not change during the application and it continued to oscillate phase-locked to its home ganglion’s PS bursts. As the membrane potential did not change, this factor could be excluded as influence on EPSP size here. Amplitude and shape of all EPSPs were similar to control in normal saline (N = 6). Similarly, application of PTX had no effect on EPSP size or shape (Fig 4B; N = 2; S4 Table). We could therefore exclude glutamate and GABA as neurotransmitters at the synapse between the Coordinating Neurons and ComInt 1, despite the puzzling effect their antagonists had on the coordination between segments.

### Coordinating Neurons are not serotonergic

In lobster, Beltz and Kravitz [19] described two medial (MFB) and lateral fiber bundles (LFB) with 5-HT-immunoreactive labeling that projected into the LN of each abdominal ganglion, the site of the swimmeret CPG connections in crayfish. Beltz and Kravitz did not identify the origins of all 5-HT-immunoreactive fiber bundles. Because of the branching in the LN and two fiber bundles running close to the midline we hypothesized that the Coordinating Neurons might be serotonergic.

Electrophysiological identification of neurotransmitters usually requires stable intracellular recordings over long periods of time to allow thorough application and washing of suitable, specific agonists and antagonists. In some cases, deteriorating recording quality can lead to misinterpretation of results, especially when focusing on low-amplitude, passively propagated EPSPs. Hence, our second approach to identify putative neurotransmitters produced by specific neurons was double labeling with intracellular dye application in the neuron of interest and immunohistochemistry using antibodies against a suspected neurotransmitter, in this case 5-HT.

**Fig 5:**
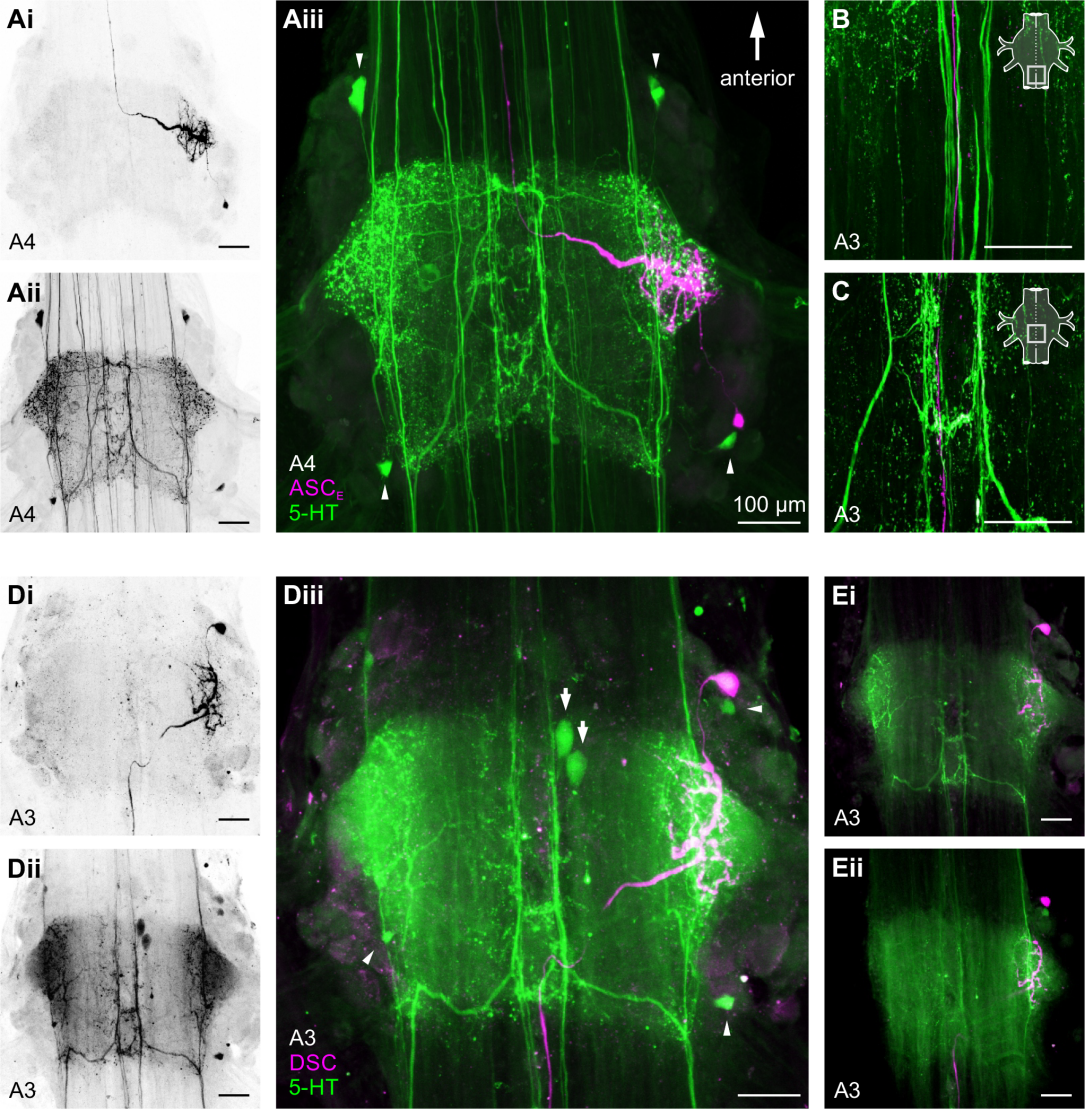
The Coordinating Neurons were not 5-HT-immunoreactive. **A:** Dorsal view of a whole-mount preparation with intracellular staining of ASC_E_ (**Ai**) and 5-HT antibody labeling (**Aii**) in A4. Merge shows no co-labeling for dye-filled ASC_E_ (magenta) and 5-HT-immunoreactive neurons and fiber bundles (green) in ASC_E_’s home ganglion (A4, **Aiii**) and one of ASC_E_’s target ganglia (A3, **B** and **C**; the area shown is indicated in the schematic). **D:** Dorsal view of a whole-mount preparation with intracellular staining of DSC (**Di**) and 5-HT antibody labeling (**Dii**) in A3. Merge shows no co-labeling for dye-filled DSC (magenta) and 5-HT-immunoreactive neurons and fiber bundles (green) in DSC’s home ganglion (**Diii**). **E:** Soma (**Ei**) and axon (**Eii**) of the Coordinating Neuron were located in different planes compared to 5-HT-immunoreactive neurons and fiber bundles. Any apparent double-labeling in the figures is due to the 2D z-stack projection (10μm step size). Scale bar (100μm) applies to all figures. Arrowheads indicate small lateral 5-HT-immunoreactive somata; arrows indicate large ventral 5-HT-immunoreactive somata. For better view please also see supplementary movies of the confocal scans of these whole mounts (S1 Video, S2 Video) A: Abdominal ganglion; ASC_E_: Ascending Coordinating Neuron; DSC: Descending Coordinating Neuron.

Like in lobster, two MFBs and two LFBs were present in all investigated ganglia (N = 17, Fig 5, S1 Video and S2 Video). LFBs branched in the area of the LN. At the posterior border of the ganglion’s core region, fibers crossed between the MFBs and LFBs. Additionally, MFBs crossed to the contralateral side at the ganglion’s mid region. Four small cell bodies were present in the lateral ventral region: two anterior and two posterior (Fig 5, arrowheads in Aiii and Diii). Depending on the preparation, up to three large cell bodies could be located ventrally at the midline (Fig 5, arrows in Diii; S1Ai, S1Bi and S1Ci Fig).

ASC_E_’s and DSC’s somata were located very close but dorsal to the posterior or anterior small 5-HT-immunoreactive cell bodies, respectively (Fig 5A and 5D, S1 Fig, S1 Video, S2 Video). Both ASC_E_’s and DSC’s dendrites branched in the 5-HT-immunoreactive neuropil region and the axons projected dorsally in close proximity to the 5-HT-immunoreactive MFBs along the midline. Nevertheless, we could not observe any co-labeling of 5-HT-immunoreactive and the Coordinating Neurons in their home ganglion (Fig 5A, 5D and 5E) or target ganglia (Fig 5B and 5C) (ASC_E_: N = 7; DSC: N = 9). If Coordinating Neurons released 5-HT at their synapse to ComInt 1 we would have expected co-labeling along the coordinating axons at the midline of their target ganglia. But the Coordinating Neurons’ somata and axons were located in different planes than the 5-HT-immunoreactive fibers (Fig 5E, S1 Fig, S1 Video, S2 Video). Therefore, we could also exclude 5-HT as a neurotransmitter of the Coordinating Neurons. Any apparent double-labeling in the figures is due to the 2D z-stack projection (see also S1 Video and S2 Video).

### Both Coordinating Neurons contain ACh

With the elimination of glutamate, GABA, and 5-HT, we could narrow down the list of putative neurotransmitters. Antibodies are very specific to their epitopes and usually designed for vertebrates. Hence, some structures in invertebrates cannot be labeled easily with standard antibodies. To continue our neurotransmitter screening and ascertain whether ACh could be a neurotransmitter of the Coordinating Neurons, we used the highly sensitive and robust method of single cell profiling by matrix-assisted laser desorption/ionization (MALDI)–time of flight (TOF) mass spectrometry.

While a MALDI-TOF MS-based method for the detection of neuropeptides on the single cell level has been established (e.g. [46]), an equivalent approach for the detection of smaller neurotransmitters such as ACh from individual dissected single cells is lacking so far. In our study, we modified a strategy published from Persike and colleagues [47] to fill this gap. The advantage of detecting ACh with MALDI-TOF is that, compared to other neurotransmitters, no further derivatizing steps are necessary because the molecule is positively charged.

**Fig 6:**
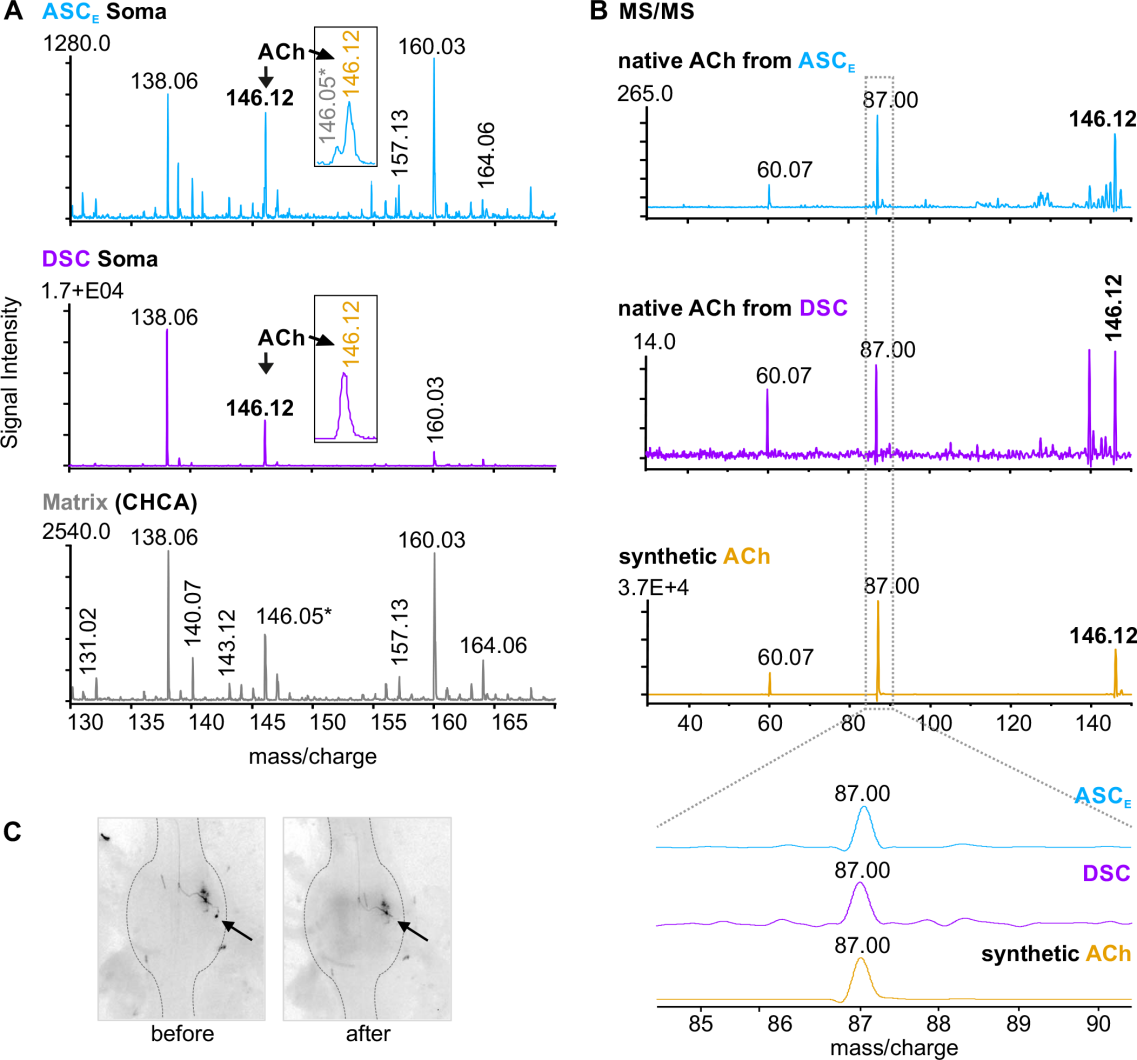
MALDI-TOF MS revealed ACh in ASC_E_ and DSC somata. **A:** Representative mass spectra from a single ASC_E_ (upper panel, cyan) and DSC soma preparation (middle panel, violet). In both, an ion signal at m/z 146.12 was detectable, which is clearly distinct from the ion signal at m/z 146.05 assigned to the matrix of cyano-4-hydroxycinnamic acid (CHCA, grey) (lower panel and *inset* in ASC_E_ panel). **B:** Tandem mass spectrometry (MS/MS) experiments confirmed the ion at m/z 146.12 (arrows in A) as ACh from ASC_E_’s (upper panel, cyan) and DSC’s soma preparations (middle panel, violet). As control MS/MS experiments were performed for synthetic ACh (lower panel, orange). **C:** Image series of a single ASC_E_ soma (arrow) dissection. ASC_E_: Ascending Coordinating Neuron; DSC: Descending Coordinating Neuron.

We dissected individual ASC_E_ (N = 5) and DSC (N = 3) somata using a glass capillary after filling the neuron with fluorescent dye (Fig 6C). For both cell types, we found an ion signal at m/z 146.12 in the resulting mass spectra, which corresponds to the theoretical mass of ACh (Fig 6A). To verify the structure of the putative ACh, the same sample spots were used for tandem mass spectrometry (MS/MS, Fig 6B) experiments. Resulting mass spectra generated an ion pattern of two distinct ion signals at m/z 60.07 and m/z 87.00, which corresponds to ACh data provided by the metabolite database METLIN. For experimental structure validation we confirmed our findings with MS/MS experiments using synthetic ACh (Fig 6B).

**Fig 7:**
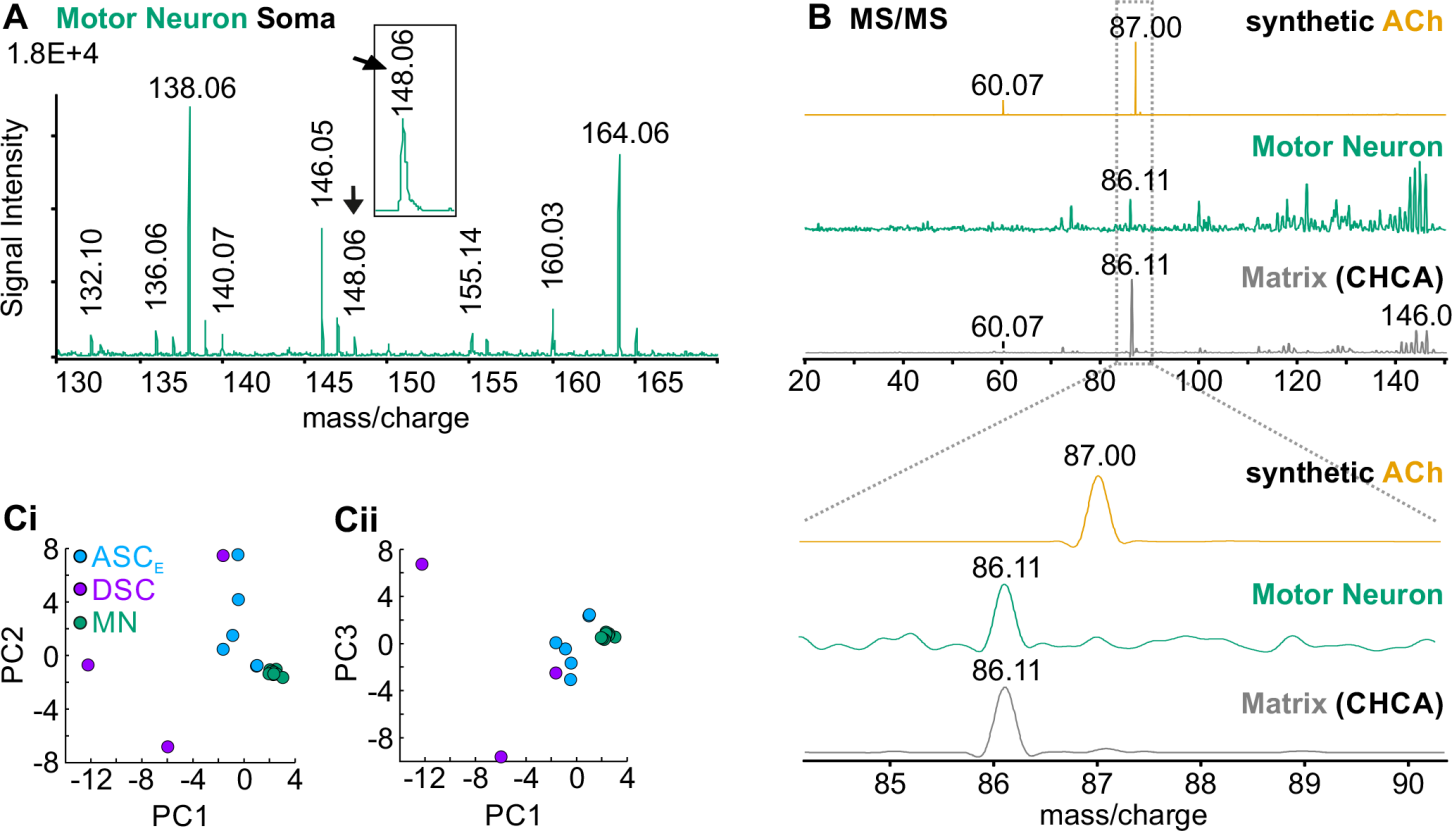
MALDI-TOF MS of individual non-cholinergic motor neuron was negative for ACh. **A:** Mass spectrum of a single motor neuron soma (green). *Inset* illustrates ion signal at m/z 146.06. **B:** Fragments of this ion (arrows in A) corresponded to CHCA (grey) and not to fragments generated from synthetic ACh (orange). **C:** Principal Component Analysis of mass spectra from individual soma preparations of ASC_E_ (N = 5, cyan), DSC (N = 3, violet), and motor neurons (n = 9, all from different ganglia from three different animals, green). The first three principal components (PC) are shown. Motor neurons formed a distinct cluster which is separate from Coordinating Neurons for PC1 vs. PC2 (**Ci**) and PC1 vs. PC3 (**Cii**). ASC_E_: Ascending Coordinating Neuron; DSC: Descending Coordinating Neuron; MN: Motor neuron.

To screen for contaminations during the dissection procedure of ASC_E_s and DSCs, we dissected individual somata from motor neurons, which are glutamatergic or GABAergic and do not synthesize ACh, as control [17, 18, 48, 49]. In all preparations (N = 3; n = 9), resulting mass spectra did not show an ion signal at m/z 146.12 but at m/z 146.06 (Fig 7A). Fragmentation experiments demonstrated that the ion signal at m/z 146.06 corresponded to CHCA, which was used as matrix (Fig 7B). Therefore, we conclude that both ASC_E_ and DSC contain ACh.

To determine the differences in the molecular composition of Coordinating Neurons and motor neurons, we performed a principal component analysis (Fig 7C). Mass spectra of individual motor neurons contained the most homogenous group of data points and formed a distinct cluster, followed by ASC_E_ data. The most variable data were obtained from the three individual DSC somata. The differences in the cluster spread of ASC_E_ and DSC could result from differences in the molecular composition of these two neuron types. However, both ASC_E_ and DSC were clearly separated from the motor neuron cluster.

With these results, we demonstrated that there was no cross contamination of the sample with ACh positive tissue that surrounded the extracted neurons, since it is known that the motor neurons use glutamate and GABA at their neuromuscular end plate [17,25,48]. Therefore, this strengthened our result that the Coordinating Neurons ASC_E_ and DSC indeed contain ACh, which they might release at the synapse to ComInt 1.

## Discussion

Using a combination of electrophysiology, immunohistochemistry, and mass spectrometry, we investigated candidate neurotransmitters of Coordinating Neurons in the crustacean central nervous system. Each of the above described methods is suitable for the investigated neurotransmitters to different extents. The limitation of electrophysiology, in this case intracellular recordings from the neuron’s primary neurite, is to maintain high quality recordings for long periods of time. Recording quality is crucial for analysis of EPSP shapes because as they are passively propagated through the neurite increased leak conductance caused by electrode penetration can drastically alter EPSP appearance.

In contrast, neuronal intophoretic dye injection and subsequent antibody labeling do not require prolonged intracellular recordings. Commercially available antibodies, however, do not always work well across species. For example, polyclonal antibody labeling of choline acetyltransferase failed in crayfish [6] (see discussion below). Mass spectrometry (MALDI-TOF) can be used to detect multiple molecules in the same sample, but molecules need to be charged. As the majority of our preselected transmitters were covalently linked, we would have needed additional steps for molecule derivatization. Only with combining the aforementioned techniques, we were able to screen for and identify particular neurotransmitters associated with specific neurons.

Electrophysiological experiments demonstrated mainly chemical synapses between the Coordinating Neurons ASC_E_ and DSC, and their postsynaptic neuron ComInt 1. Neither bath nor focal application of glutamatergic and GABAergic antagonists caused significant changes in the activity of the postsynaptic ComInt 1 or changes in the shape and size of EPSPs that are elicited by the Coordinating Neurons’ spikes. Thus, Coordinating Neurons do not release glutamate or GABA at their synapse to ComInt 1. Using immunohistochemistry, we could additionally exclude 5-HT as the neurotransmitter of ASC_E_ and DSC. However, using single cell analysis by MALDI-TOF mass spectrometry, at the level of single identified neurons, we could identify ACh as most likely neurotransmitter of the Coordinating Neurons.

EPSPs elicited by the two neighboring Coordinating Neurons (ASC_E_ and DSC) that synapse on each ComInt 1 have distinct amplitudes but similar kinetics [14]. Low Ca^2+^ / high Mg^2+^ saline effectively blocks neurotransmitter release at chemical synapses in the crayfish swimmeret system [8]. Here we demonstrated that EPSPs were still elicited in ComInt 1 but their amplitude was on average reduced by 50% in low Ca^2+^ / high Mg^2+^ saline. It is possible that EPSPs in ComInt1 were not completely blocked while using low Ca^2+^ / high Mg^2+^ saline, e.g. Weller and colleagues demonstrated that calcium concentration non-linearly affected crustacean muscle fiber EJPs [49]. Additionally, the recording of ComInt 1 was not conducted at the surface of the ganglion, thus some calcium-ions may have been present in the deeper tissue. These slightly elevated levels may have been sufficient to cause the release of some neurotransmitter vesicles from the presynaptic Coordinating Neurons, eliciting small EPSPs. Alternatively, the synapse between Coordinating Neurons and ComInt 1 may have a small electrical component, which would explain why EPSPs were still elicited in low Ca^2+^ / high Mg^2+^ saline.

In our electrophysiological and immunohistochemical attempts to identify the neurotransmitter of ASC_E_ and DSC, we investigated the effect of glutamate and GABA antagonists, as well as 5-HT, on the coordination of the swimmeret system. Although all of the tested substances had an effect on the system’s motor output, neither PTX nor DNQX affected the EPSPs in ComInt 1. Motor activity was clearly rhythmic in DNQX, whereas in PTX the activity appeared tonic with rhythmic modulation. This indicates that PTX disinhibited motor neurons, which still received rhythmic inhibition by the CPG. With regard to our current knowledge, CPG neurons are the only neurons to inhibit motor neurons [35]. Hence, it seems likely that the CPG neurons use GABA as neurotransmitter. This is in accordance with results from Sherff and Mulloney [25], who demonstrated that both GABA and glutamate affect the motor neurons.

Antibody labeling against 5-HT demonstrated that the Coordinating Neurons were not 5-HT-immunoreactive. Based on these results we could exclude glutamate, GABA, and 5-HT as neurotransmitters of the Coordinating Neurons although all of them influenced the motor output. This indicates that coordination can be interrupted at other levels besides the interpretation of arriving coordinating information.

Cholinergic neurons are present throughout the crustacean central nervous system. However, analysis of nervous tissue extracts from sensory fibers show considerable higher choline acetyltransferase activity and ACh content than extracts from nerves containing motor axons only, and the transferase activity is higher in central ganglia compared to connectives [49–51]. When Barker and colleagues [51] severed sensory roots from ganglia, newly synthesized ACh was greatly reduced in those ganglia. Although ACh is mostly associated with sensory neurons in decapod crustaceans [37, 52], the neurotransmitter is also used by certain motor neurons in the lobster stomatogastric system [29, 53, 54]. For decades, cholinergic agonists have been used to activate CPGs and elicit activity from isolated crustacean nervous preparations, e.g. the walking system or swimmeret system [20, 28, 55, 56]. But so far, no cholinergic neurons have been identified in the circuit of the swimmeret system. Here we present evidence by mass spectrometry that ACh is present in the coordinating interneurons (Fig 6), identifying one possible source of cholinergic input in the system.

Mapping of high ACh esterase activity in the crayfish abdominal ganglia by Braun and Mulloney [57] labeled neurons in the LN, the area of PS and RS somata clusters, and interganglionic neurons. The Coordinating Neurons are also located in these areas. Additionally, Braun and Mulloney detected labeled axons in the Minuscule Tract, which comprises only eight axons, among them the Coordinating Neurons’ axons [8]. A more reliable mapping of cholinergic neurons would be to label the synthesizing enzyme choline acetyltransferase instead of visualizing esterase activity but polyclonal antibodies against the transferase has failed so far [6]. In our study, we used single cell analysis for ACh recognition in the Coordinating Neurons. MS/MS experiments of the putative ACh ion signals at m/z 146.12 showed a characteristic fragmentation pattern originating from the loss of protonated trimethylamine (m/z 60.07) from the residual C_4_H_7_O_2_^+^ ion (m/z 87.00) by a neighboring group attack [58].

In single-cell mass spectrometry, dissected somata could have been contaminated by attached surrounding tissue or axons. To verify our single-cell dissection protocol and thus exclude false-positive results for the Coordinating Neurons, we additionally dissected non-cholinergic motor neurons, which are surrounded by cholinergic-positive tissue and are located in the same areas as ASC_E_ and DSC somata. All these control experiments were negative for ACh so that our findings provide strong evidence for ACh as the neurotransmitter of the Coordinating Neurons. This corroborates previous findings from Mulloney and Hall [23], who demonstrated in split-bath experiments that simultaneous application of different concentrations of carbachol to each half of the preparation altered the phase relationships at the boundary between segments bathed in high and low carbachol concentration. Experiments in which the Coordinating Neurons were electrically stimulated led to similar changes in phase relationships as those split-bath experiments [11, 23]. This previous result already indicated that ACh is engaged in the coordination.

The only identified neuron postsynaptic to ASC_E_ and DSC is ComInt 1 [14]. Now that we have evidence of the neurotransmitter, we can investigate the functional implications in future studies. Cholinergic agonists are known to affect the swimmeret system via two pathways [20]. Muscarine or its agonists activate the swimmeret rhythm in quiescent preparations and have only a minor dose-dependent effect on the rhythm’s frequency. In contrast, activating nicotinic receptors markedly increases frequency in a dose-dependent fashion but does not elicit activity from a quiescent preparation. Manipulating ACh levels and observing how this changes the interpretation of coordinating information helps to answer the question of how the swimmeret system maintains its strictly coordinated pattern even if cycle period is changing [23]. With this new knowledge, we will be able to pursue detailed studies of how modifying such information can change coordination across multiple neuronal oscillators.

## Supporting information

S1 Fig Coordinating Neurons and 5-HT-immunoreactive neurons were not co-labeled. Depth coded anti-5-HT antibody labeling (**Ai, Bi, Ci**) or Coordinating Neuron staining (**Aii, Bii, Cii**). Dorsal regions are shown in red while ventral ones are blue. A and B are the depth coded projections from Fig 5A and 5D. **C:** ASC_E_ and DSC were dye-filled in the same hemiganglion. The large 5-HT-immunoreactive cells were located more ventrally (blue shades) than the somata of the Coordinating Neurons (green or cyan shades). The 5-HT-immunoreactive LFB and MFB were located in the middle of the ganglion (green shades) while the Coordinating Neurons projected more dorsally (red shades).
(TIF)

S1 Table Phase data and statistics for ipsilateral power-strokes for blocking experiments. (XLSX)

S2 Table EPSP amplitudes in ComInt 1 in normal saline and low Ca^2+^ / high Mg^2+^ saline. (XLSX)

S3 Table Shape indices and statistics of EPSPs elicited by ASC_E_ or DSC in two different ComInt 1s. (XLSX)

S4 Table Shape indices and statistics of EPSPs for blocking experiments. (XLSX)

S1 Video Co-labeling of ASC_E_ and 5-HT fiber bundles. This video shows a dorsal-to-ventral progression through the z-stack to demonstrate that ASC_E_ is not co-localized with any of the 5-HT-immunoreactive fiber bundles. ASC_E_ is shown in magenta, 5-HT antibody labeling in green.
(AVI)

S2 Video Co-labeling of DSC and 5-HT fiber bundles. This video shows a dorsal-to-ventral progression through the z-stack to demonstrate that DSC is not co-localized with any of the 5-HT-immunoreactive fiber bundles. DSC is shown in magenta, 5-HT antibody labeling in green.
(AVI)
