# Supplementary figures and images for: Profiling neurotransmitters in a crustacean neural circuit for locomotion
Source: PLoS One. 2018 May 22;13(5):e0197781. doi: 10.1371/journal.pone.0197781 (PMC5963771; doi:10.1371/journal.pone.0197781)

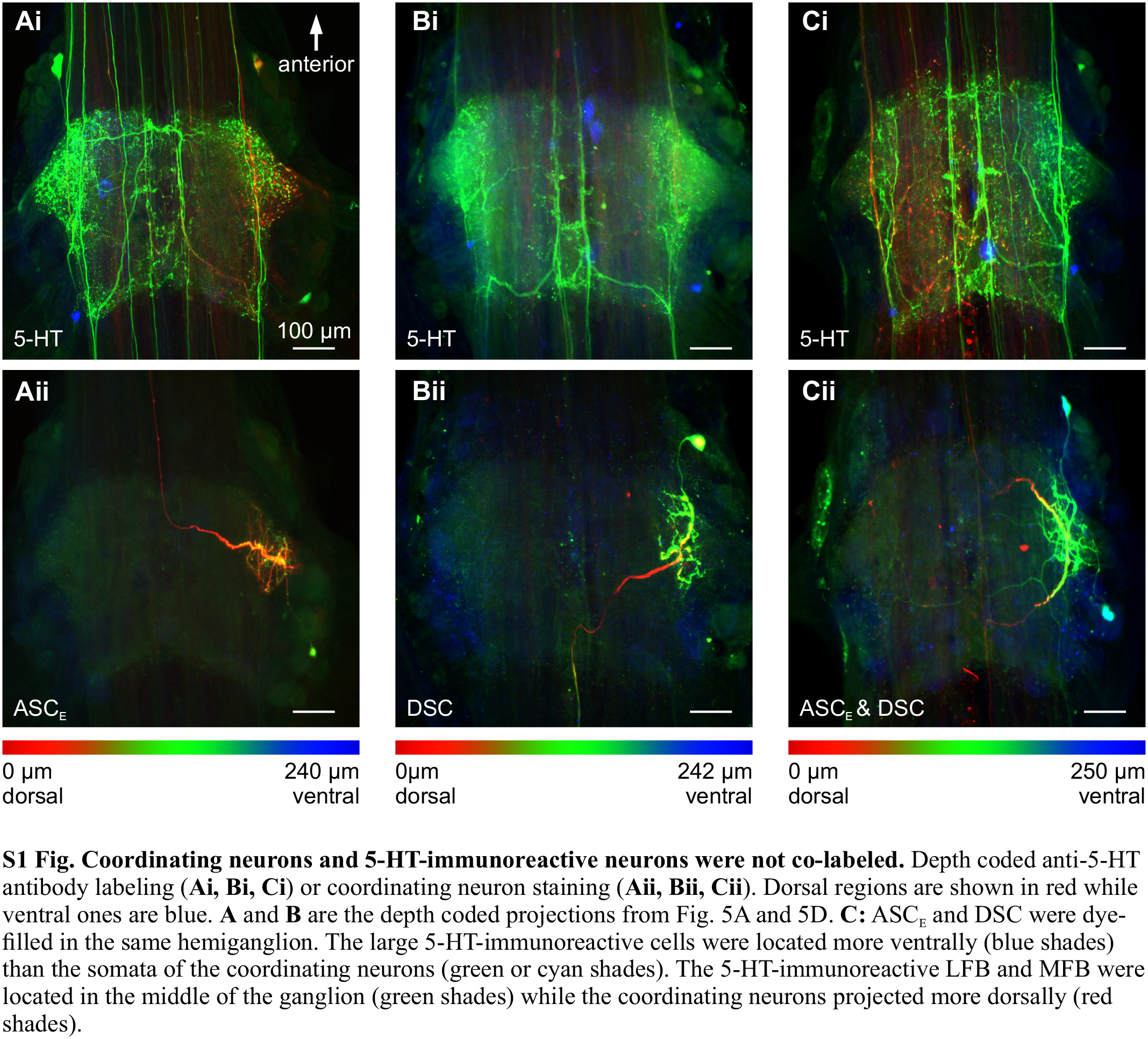

Supplement: S1 Fig — Depth coded anti-5-HT antibody labeling (Ai, Bi, Ci) or Coordinating Neuron staining (Aii, Bii, Cii). Dorsal regions are shown in red while ventral ones are blue. A and B are the depth coded projections from Fig 5A and 5D. C: ASCE and DSC were dye-filled in the same hemiganglion. The large 5-HT-immunoreactive cells were located more ventrally (blue shades) than the somata of the Coordinating Neurons (green or cyan shades). The 5-HT-immunoreactive LFB and MFB were located in the middle of the ganglion (green shades) while the Coordinating Neurons projected more dorsally (red shades). (TIF) [file pone.0197781.s001.tif]
